# Mitofusin 2 Participates in Mitophagy and Mitochondrial Fusion Against Angiotensin II-Induced Cardiomyocyte Injury

**DOI:** 10.3389/fphys.2019.00411

**Published:** 2019-04-10

**Authors:** Wenjun Xiong, Zhuang Ma, Dongqi An, Zuheng Liu, Wanqiang Cai, Yujia Bai, Qiong Zhan, Wenyan Lai, Qingchun Zeng, Hao Ren, Dingli Xu

**Affiliations:** ^1^State Key Laboratory of Organ Failure Research, Department of Cardiology, Nanfang Hospital, Southern Medical University, Guangzhou, China; ^2^Key Laboratory for Organ Failure Research, Ministry of Education of the People’s Republic of China, Guangzhou, China; ^3^Department of Rheumatology, Nanfang Hospital, Southern Medical University, Guangzhou, China

**Keywords:** MFN2, mitochondria, autophagy, angiotensin II, ROS

## Abstract

**Background:**

Mitochondrial dynamics play a critical role in mitochondrial function. The mitofusin 2 (MFN2) gene encodes a mitochondrial membrane protein that participates in mitochondrial fusion to maintain and operate the mitochondrial network. Moreover, MFN2 is essential for mitophagy. In Ang II-induced cardiac remodeling, the combined effects of MFN2-mediated mitochondrial fusion and mitophagy are unclear. This study was designed to explore a novel strategy for preventing cardiomyocyte injury via modulation of mitochondrial dynamics.

**Methods:**

We studied the function of MFN2 in mitochondrial fusion and mitophagy in Ang II-stimulated cardiomyocyte injury. Cardiomyocyte injury experiments, including reactive oxygen species (ROS) production, mitochondrial membrane potential (MMP), and apoptosis rate of cardiomyocytes were performed. The mitochondrial morphology in cardiomyocytes was examined via transmission electron microscopy (TEM) and confocal microscopy. Autophagic levels in response to Ang II were examined by immunoblotting of autophagy-related proteins. Moreover, PINK1/MFN2/Parkin pathway-related proteins were examined.

**Results:**

With stimulation by Ang II, MFN2 expression was progressively reduced. MFN2 deficiency impaired mitochondrial quality, resulting in exacerbated mitochondrial damage induced by Ang II. The Ang II-induced increases in ROS production and apoptosis rate were alleviated by MFN2 overexpression. Moreover, MFN2 alleviated the Ang II-induced reduction in MMP. MFN2 promoted mitochondrial fusion, and MFN2 promoted Parkin translocation and phosphorylation, leading to mitochondrial autophagy. The effects of MFN2 overexpression were reversed by autophagy inhibitors.

**Conclusion:**

Mitofusin 2 promotes Parkin translocation and phosphorylation, leading to mitophagy to clear damaged mitochondria. However, the beneficial effects of MFN2 were reversed by autophagy inhibitors. Additionally, MFN2 participates in mitochondrial fusion to maintain mitochondrial quality. Thus, MFN2 participated in mitophagy and mitochondrial fusion against Ang II-induced cardiomyocyte injury.

## Introduction

Ventricular remodeling is the core foundation for the development of heart failure (Hill and Olson, 2008). Pathological remodeling is typically due to a number of causes that result in increased pressure or volume, causing pressure or volume overload in the heart (Burchfield et al., 2013). This pathological process may include ventricular hypertrophy, ventricular dilation, cardiomegaly, and other changes. Ultimately, ventricular remodeling results in heart failure. Neurohumoral stimulation and mechanical stress are the major triggers of ventricular remodeling. Ang II can activate a number of signaling pathways to induce cardiac inflammation, hypertrophy, and fibrosis, thus leading to ventricular remodeling (Mehta and Griendling, 2007; Trachtenberg and Hare, 2017). Additionally, ventricular remodeling involves mitochondrial metabolism, oxidative stress, and autophagy (Maillet et al., 2013; van Berlo et al., 2013; Zhou et al., 2013; Lyon et al., 2015).

In recent years, insufficient myocardial energy has been considered one of the mechanisms underlying the occurrence and development of ventricular remodeling. As a site of energy synthesis, mitochondria are closely related to the development of heart failure. Thus, the balance of the mitochondrial network has been recognized as a critical factor in maintaining cellular energy homeostasis and cardiac function. Mitochondria are highly dynamic organelles, and their morphology is determined by the equilibrium between mitochondrial fusion and fission events. The physiological function of mitochondria depends on the tightly connected mitochondrial network. The maintenance of this mitochondrial network is mainly controlled by mitochondrial fusion and fission proteins (Cao et al., 2017). The MFN2 gene encodes a mitochondrial membrane protein that participates in mitochondrial fusion and contributes to the maintenance and operation of the mitochondrial network (Santel and Fuller, 2001; Rojo et al., 2002; Cao et al., 2017). Moreover, MFN2 is involved in the clearance of damaged mitochondria via selective autophagy (Chen and Dorn, 2013). Autophagy is a regulated mechanism leading to the disassembly of dysfunctional proteins or organelles and the removal of intracellular pathogens (Klionsky, 2008; Levine and Kroemer, 2008). Autophagy also enables the orderly degradation and recycling of cellular components (Melser et al., 2015). Thus, understanding the balance of mitochondrial network regulation may facilitate the discovery of a novel strategy to combat heart failure.

Evidence has revealed that MFN2 participates in mitochondrial quality control. Some previous studies have shown that the accumulation of morphologically and functionally abnormal mitochondria induced respiratory dysfunction in MFN2-deficient mice, causing dilated cardiomyopathy (Chen and Dorn, 2013). Thus, MFN2 is required for quality control of cardiac mitochondria. Although these data suggest that MFN2 plays a central role in the balance of mitochondria. In Ang II-induced cardiac remodeling, the combined effects of MFN2-mediated mitochondrial fusion and mitophagy are unclear. Therefore, we hypothesized that MFN2 can protect cardiac function against Ang II-induced cardiomyocyte injury. We overexpressed MFN2 *in vitro* to study the effects of MFN2 on cardiac function, mitochondrial fusion and mitophagy. We found that MFN2 maintained mitochondrial quality through participating in mitophagy and mitochondrial fusion in an Ang II-induced cardiomyocyte injury model.

## Materials and Methods

### Chemicals and Reagents

Fetal bovine serum (FBS) and Dulbecco’s Modified Eagle’s Medium (DMEM) were purchased from HyClone (Logan, UT, United States). Trypsin and collagenase were purchased from Sigma-Aldrich Co. (Saint Louis, MO, United States). Ang II and chloroquine diphosphate salt (CQ) was purchased from Sigma-Aldrich Co., Carbamide was purchased from Solarbio Science & Technology Co., Ltd. (Beijing, China). The following polyclonal primary antibodies were used in this study: anti-MFN2, anti-Parkin, anti-P-Parkin, anti-PINK1, anti-DRP1, anti-Beclin1, anti-P62, anti-GAPDH, anti-COX-IV, anti-β-actin (Abcam, Cambridge, United Kingdom), and anti-LC3B (Sigma-Aldrich Co.).

### Cell Culture and Adenoviral Transduction

Neonatal rat cardiomyocytes were prepared from the hearts of 1- to 3-day-old Sprague-Dawley rats using enzymatic dissociation and cultured as previously described (Xiong et al., 2018). Recombinant adenoviruses for MFN2 overexpression (Ad-MFN2) were designed and synthesized by GeneChem Co., Ltd. (Shanghai, China). The viruses were added to cells according to the manufacturer’s instructions. After adenoviral transduction for 72–96 h, cells were stimulated with 1 μM Ang II for 24 h for subsequent experiments and analyses.

### Measurement of the Reactive Oxygen Species (ROS) Levels

For the detection of intracellular superoxide anion, dihydroethidium (DHE, Invitrogen Molecular Probes, United States) was used to evaluate intracellular ROS production according to the manufacturer’s instructions. Briefly, the cells were incubated with culture medium containing 10 μM DHE at 37°C for 30 min for fluorescent probe loading, followed by three washes with phosphate-buffered saline (PBS). Cells were stained with Hoechst for 10 min. Then, ROS production was examined with a fluorescence microscope (Olympus, Japan). Bright red fluorescence represents ROS concentration, and blue fluorescence represents cell nuclei. ImageJ was used for analysis.

### Measurement of MMP

JC-1 staining assay was performed using MMP detection according to the manufacturer’s instructions. Briefly, cardiomyocytes were incubated with JC-1 staining solution at 37°C for 20 min and then washed three times with JC-1 staining buffer. Fluorescent cardiomyocytes were observed by a fluorescence microscope (Olympus, Japan). Bright red fluorescence represents J-aggregate and green fluorescence represents J-monomer. ImageJ was used for analysis.

### TUNEL Assay

The apoptosis rate of cardiomyocytes was examined using a TdT-mediated dUTP nick-end labeling (TUNEL) assay (One Step TUNEL Apoptosis Assay Kit; Beyotime, Jiangsu, China) according to the manufacturer’s instructions. Cardiomyocytes were fixed in 4% paraformaldehyde for 30 min and permeabilized in 0.3% Triton X-100 for 5 min. Then, the cells were incubated with TUNEL staining solution at 37°C for 30 min and were then washed three times with PBS. Cells were stained with DAPI for 10 min to counterstain the nuclei. Then, TUNEL-positive cells were examined with a fluorescence microscope (Olympus, Japan). Bright green fluorescence represents TUNEL-positive cells, and blue fluorescence represents cell nuclei. ImageJ was used for analysis.

### Detection of the Colocalization of Mitochondria and Lysosomes

The colocalization of mitochondria and lysosomes indirectly indicated mitophagy. Cells were coincubated with LysoTracker Red (50 nM) and MitoTracker Green (a fluorescent probe for mitochondria that does not depend on MMP, 100 nM, Molecular Probes, Eugene, OR, United States) for 30 min. Then, cells were stained with Hoechst for 10 min followed by three washes with PBS. Fluorescent images were observed with a Zeiss LSM 880 (Zeiss, Germany). Bright green fluorescence represents mitochondria, bright red fluorescence represents lysosomes, and blue fluorescence represents cell nuclei. The number of MitoTracker- and LysoTracker-positive foci indirectly indicated mitophagy.

### Transmission Electron Microscopy

Mitochondrial morphology was observed by transmission electron microscopy (TEM). Small fragments of cardiomyocytes (1 mm^3^ in size) were fixed by immersion in 2.5% glutaraldehyde in cacodylate buffer (0.1 M, pH 7.4) for 2 h. The fragments were rinsed three times in PBS for 10 min each time and then postfixed in 1% osmium tetroxide in cacodylate buffer (0.1 M. pH 7.4) for 2 h. After being rinsed in cacodylate buffer, the fragments were subsequently rinsed three times in PBS for 10 min each time. Then, the fragments were dehydrated in graded ethanol (50, 70, 90, and 100%), rinsed with propylene oxide, permeabilized with a 1:1 mixture of propylene oxide and Epon 812, and then baked in a 38°C oven for 3 h. Then, the above steps were repeated using a 1:2 mixture of propylene oxide and Epon 812. Ultrathin sections (80 nm) were cut with an ultramicrotome (UC7, Leica, Germany), contrast stained with uranyl acetate and lead citrate and examined with an electron microscope (JEM-1400, Japan) operated at 80 kV.

### Mitochondria Isolation

The cardiomyocytes were digested with trypsin, homogenized with a homogenizer, and then centrifuged at 600 ×*g* for 10 min at 4°C. The supernatant was centrifuged at 11,000 ×*g* for 10 min at 4°C, and the remaining supernatant was discarded. The bottom precipitate was the mitochondria. Mitochondrial lysis buffer (Beyotime, Jiangsu, China) was added to the precipitate to extract mitochondrial proteins for subsequent experiments and analyses.

### Immunoblot Analysis

For total protein extraction, the cells were lysed in lysis buffer (Beyotime, Jiangsu, China). Cytoplasmic and mitochondrial proteins were extracted from total proteins using a Cell Mitochondria Isolation Kit (Beyotime, Jiangsu, China). In order to obtain insoluble p62 protein, carbamide was added to the cell lysate. Western blot analysis was performed as previously reported (Xiong et al., 2018). The density of the expressed protein bands was quantified by ImageJ/Fiji software (NIH, Bethesda, MD, United States).

### Statistical Analysis

The data are expressed as the means ± SD. Statistical analyses were performed using either one-way analysis of variance or Student’s *t*-tests (GraphPad Prism 6.0 software, San Diego, CA, Unites States). *P*-values < 0.05 were considered indicative of statistical significance.

## Results

### Ang II Induced Cardiomyocyte Injury and Decreased MFN2 Expression

**FIGURE 1:**
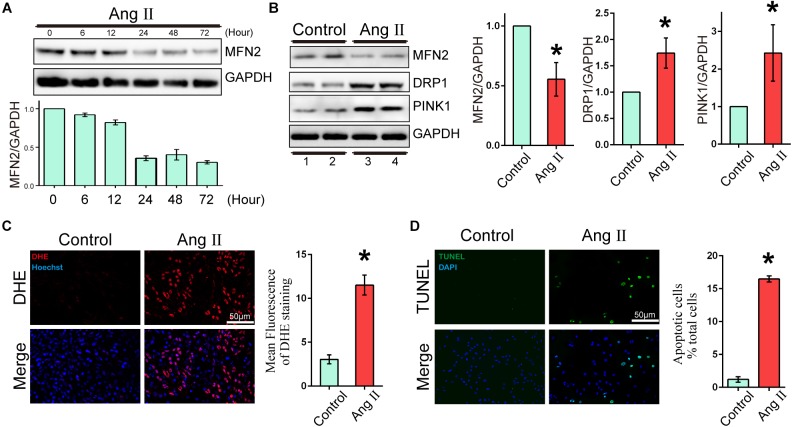
Ang II induced cardiomyocyte injury and decreased expression of MFN2. Cardiomyocytes were divided into two groups: (1) Control, cells at 80% confluence cultured without Ang II; and (2) Ang II, 1 μM Ang II added to the cell culture medium for 24 h; **(A)** Immunoblotting showing the expression of MFN2 for the indicated times. **(B)** Immunoblotting showing the expression of MFN2, DRP1, and PINK1 in cardiomyocytes. **(C)** DHE staining showing the oxidative stress activity of cardiomyocytes. DHE staining is shown in red, representing ROS production, and Hoechst staining in the nuclei is shown in blue. **(D)** Cells were stained with TUNEL. TUNEL-positive cell is shown in green, and DAPI staining in the nuclei is shown in blue. Data are presented as the means ± SD, (*n* = 3). ^∗^*P* < 0.05 vs. Control.

To evaluate the expression changes in MFN2 in AngII-stimulated cardiomyocyte models, we analyzed changes in the expression levels of MFN2, PINK1, and DRP1. Cardiomyocytes were divided into two groups: (1) Control, cells were not treated with Ang II, and (2) Ang II, cells were treated with 1 μM Ang II for 24 h. With increased AngII stimulation time, the expression of MFN2 decreased at 24 h (Figure 1A). The Western blot results of cardiac cells exposed to Ang II (1 μM) for 24 h showed that the expression levels of PINK1 and DRP1 were increased (Figure 1B and Supplementary Figure S1). ROS production was used as an indicator of cardiomyocyte injury induced by Ang II. Compared to the control condition, AngII stimulation significantly increased ROS production as shown by DHE staining, which was observed by red fluorescence intensity (Figure 1C). Additionally, AngII stimulation significantly increased cardiomyocyte apoptosis as shown by TUNEL assay (Figure 1D). Thus, 1 μM Ang II stimulation significantly induced myocardial cell injury after 24 h. These results indicated that in the Ang II model (1 μM, 24 h), the aggravation of cell injury was accompanied by the decrease in MFN2.

### MFN2 Overexpression Prevented Ang II-Induced Cardiomyocyte Injury

**FIGURE 2:**
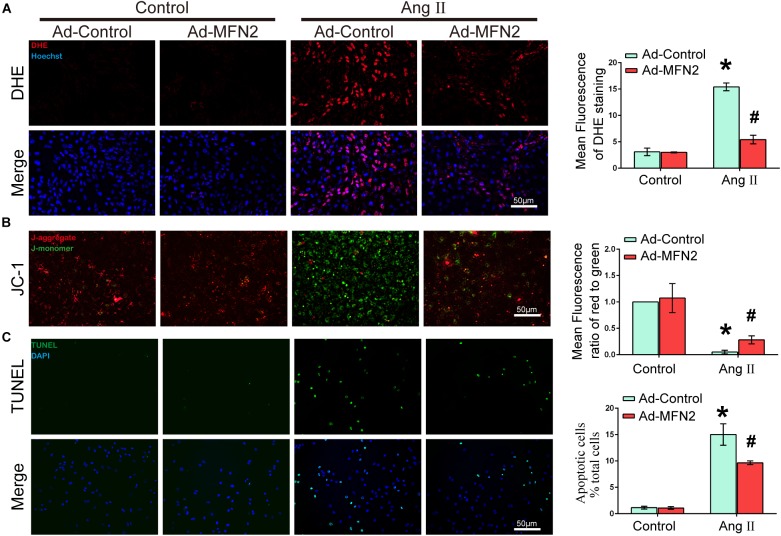
Overexpression of MFN2 prevented Ang II-induced cardiomyocyte injury. Cardiomyocytes were divided into four groups: (1) Control+Ad-Control, cells were transfected with Control Adenovirus and were not treated with Ang II; (2) Control+Ad-MFN2, cells were transfected with MFN2 Adenovirus and were not treated with Ang II; (3) Ang II+Ad-Control, cells were transfected with Control Adenovirus and treated with 1 μM Ang II for 24 h; and (4) Ang II+Ad-MFN2, cells were transfected with MFN2 Adenovirus and treated with 1 μM Ang II for 24 h; **(A)** DHE staining showing the oxidative stress activity of cardiomyocytes. DHE staining is shown in red, representing ROS production, and Hoechst staining in the nuclei is shown in blue. **(B)** Fluorescence images of cardiomyocytes stained with JC-1 tracker. J-monomer staining is shown in green, and J-aggregate staining is shown in red. **(C)** Cells were stained with TUNEL. TUNEL-positive cell is shown in green, and DAPI staining in the nuclei is shown in blue. Data are presented as the means ± SD, (*n* = 3). ^∗^*P* < 0.05 vs. Control+Ad-Control; ^#^*P* < 0.05 vs. Ang II+Ad-Control.

To evaluate the effects of MFN2 overexpression on Ang II-induced cardiomyocyte injury, we analyzed intracellular ROS production, MMP and apoptosis rate. Cardiomyocytes were divided into four groups: (1) Control+Ad-Control, cells were transfected with control adenovirus and were not treated with Ang II; (2) Control+Ad-MFN2, cells were transfected with MFN2 adenovirus and were not treated with Ang II; (3) Ang II+Ad-Control, cells were transfected with control adenovirus and treated with 1 μM Ang II for 24 h; and (4) Ang II+Ad-MFN2, cells were transfected with MFN2 adenovirus and treated with 1 μM Ang II for 24 h. The ROS product in the Ang II+Ad-MFN2 group was significantly decreased compared to that in the Ang II+Ad-Control group (Figure 2A). Additionally, after transfection with the MFN2-overexpressing adenovirus, JC-1 staining showed that the overexpression of MFN2 mitigated the Ang II-induced reduction in MMP (Figure 2B). Decreased MMP represented the early stage of apoptosis in cells. Moreover, a TUNEL assay showed that overexpression of MFN2 significantly decreased AngII-induced cardiomyocyte apoptosis (Figure 2C). These results indicated that overexpression of MFN2 prevented Ang II-induced injury, including oxidative stress, MMP and cell apoptosis rate.

### MFN2 Overexpression Enhanced Cardiomyocyte Mitophagy

**FIGURE 3:**
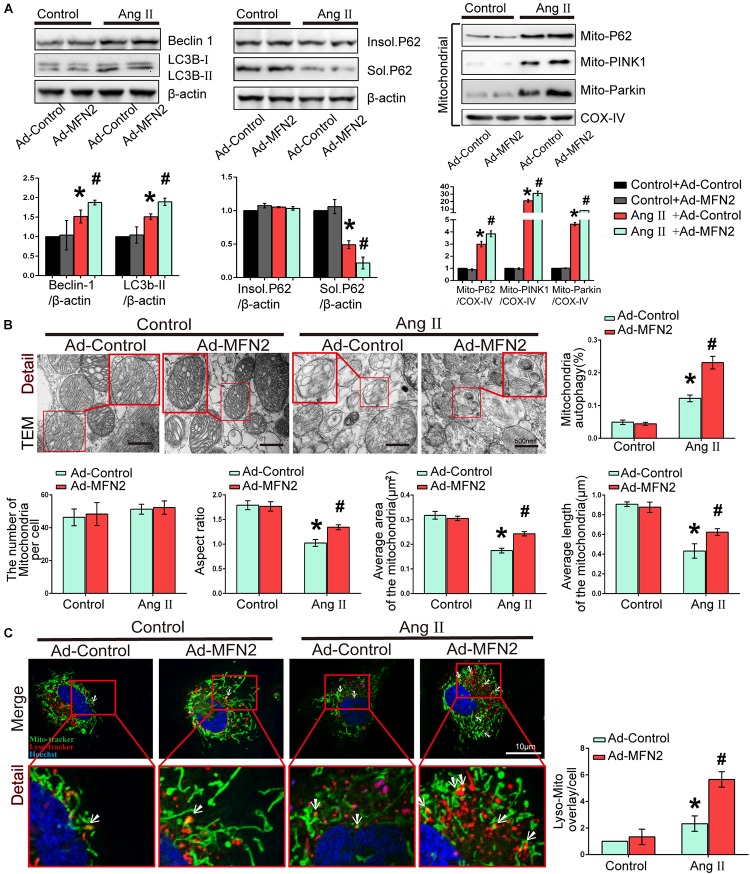
Overexpression of MFN2 enhanced cardiomyocyte mitophagy. Cardiomyocytes were divided into four groups: (1) Control+Ad-Control, cells were transfected with Control Adenovirus and were not treated with Ang II; (2) Control+Ad-MFN2, cells were transfected with MFN2 Adenovirus and were not treated with Ang II; (3) Ang II+Ad-Control, cells were transfected with Control Adenovirus and treated with 1 μM Ang II for 24 h; and (4) Ang II+Ad-MFN2, cells were transfected with MFN2 Adenovirus and treated with 1 μM Ang II for 24 h; **(A)** Immunoblotting showing the expression of Beclin1, Sol.P62, Insol.P62, and LC3B in cardiomyocytes (left) and the expression of P62, PINK1, and Parkin in the mitochondrial fraction (right). **(B)** TEM showing mitochondrial morphology in cardiomyocytes. An enlarged image in the red frame shows the details of mitochondrial structures. **(C)** Colocalization of mitochondria and lysosomes indirectly indicated mitophagy. Mitochondria are shown in green, lysosomes are shown in red, and Hoechst staining in the nuclei is shown in blue. An enlarged image in the red frame shows the details of mitochondrial. Data are presented as the means ± SD, (*n* = 3). ^∗^*P* < 0.05 vs. Control+Ad-Control; ^#^*P* < 0.05 vs. Ang II+Ad-Control.

We then investigated the effects of MFN2 overexpression on potential changes related to mitophagy in the Ang II model. Cardiomyocytes were divided into four groups: (1) Control+Ad-Control, cells were transfected with control adenovirus and were not treated with Ang II; (2) Control+Ad-MFN2, cells were transfected with MFN2 adenovirus and were not treated with Ang II; (3) Ang II+Ad-Control, cells were transfected with control adenovirus and treated with 1 μM Ang II for 24 h; and (4) Ang II+Ad-MFN2, cells were transfected with MFN2 adenovirus and treated with 1 μM Ang II for 24 h. We detected soluble p62, insoluble p62 and LC3B-I/II conversion to comprehensively determine the state of autophagy flow. The results showed that soluble p62 protein decreased, the insoluble p62 did not change significantly, and the LC3B-II/β-actin increased in the Ang II+Ad-MFN2 group compared to that in the Ang II+Ad-Control group. Moreover, We detected p62, PINK1, and Parkin in the mitochondrial fraction. The results of cardiomyocytes exposed to Ang II showed that the expression levels of Mito-P62, Mito-PINK1, and Mito-Parkin were significantly increased. MFN2 overexpression further increased the expression of Mito-P62, Mito-PINK1, and Mito-Parkin in the Ang II+Ad-MFN2 group compared to the Ang II+Ad-Control group. Thus, MFN2 overexpression increased the expression levels of autophagy-associated proteins, including Beclin1, LC3B II/soluble p62, Mito-P62, Mito-PINK1, and Mito-Parkin (Figure 3A). TEM showed that some of the mitochondria in the Control+Ad-Control group and Control+Ad-MFN2 group showed intima swelling. In the Ang II+Ad-Control group, most of the mitochondria showed swelling of the inner and outer membranes, the cytoplasm moved to the periphery, multiple focal vacuoles were present in the stroma, matrix material was lost, part of the mitochondrial swelling caused the membrane rupture, aspect ratio of the mitochondria was decreased, average area and length of the mitochondria was reduced. mitochondrial morphological disorder were ameliorated in the Ang II+Ad-MFN2 group compared to the Ang II+Ad-Control group, and mitochondrial autophagy occurred (Figure 3B). Additionally, fluorescence images of the colocalization of lysosomes and mitochondria showed that the mitochondrial and lysosomal interaction regions (white arrow) were further enhanced in the group overexpressing MFN2 compared to the control group (Figure 3C). These results indicated that overexpression of MFN2 enhanced cardiomyocyte mitophagy and improved mitochondrial quality.

### MFN2 Overexpression Enhanced Mitochondrial Fusion

**FIGURE 4:**
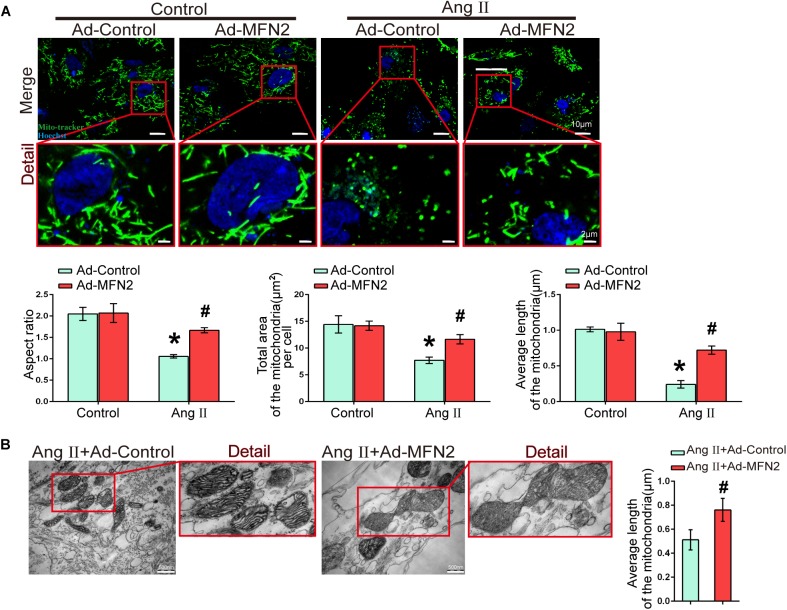
Overexpression of MFN2 enhanced cardiomyocyte mitochondrial fusion. Cardiomyocytes were divided into four groups: (1) Control+Ad-Control, cells were transfected with Control Adenovirus and were not treated with Ang II; (2) Control+Ad-MFN2, cells were transfected with MFN2 Adenovirus and were not treated with Ang II; (3) Ang II+Ad-Control, cells were transfected with Control Adenovirus and treated with 1 μM Ang II for 24 h; and (4) Ang II+Ad-MFN2, cells were transfected with MFN2 Adenovirus and treated with 1 μM Ang II for 24 h; **(A)** Mito-Tracker staining indicated mitophagy morphology. Mitochondria are shown in green, and Hoechst staining in the nuclei is shown in blue. An enlarged image in the red frame shows the details of mitochondrial structures. Each sample was quantitatively analyzed under ten random microscope views. Data are presented as the means ± SD, (*n* = 3). ^∗^*P* < 0.05 vs. Control+Ad-Control; ^#^*P* < 0.05 vs. Ang II+Ad-Control. **(B)** TEM showing mitochondrial fusion in cardiomyocytes. An enlarged image in the red frame shows the details of mitochondrial structures. Data are presented as the means ± SD, (*n* = 3). ^#^*P* < 0.05 vs. Ang II+Ad-Control.

We next evaluated the effect of MFN2 overexpression on mitochondrial fusion during myocardial injury. Cardiomyocytes were divided into four groups: (1) Control+Ad-Control, cells were transfected with control adenovirus and were not treated with Ang II; (2) Control+Ad-MFN2, cells were transfected with MFN2 adenovirus and were not treated with Ang II; (3) Ang II+Ad-Control, cells were transfected with control adenovirus and treated with 1 μM Ang II for 24 h; and (4) Ang II+Ad-MFN2, cells were transfected with MFN2 adenovirus and treated with 1 μM Ang II for 24 h. Fluorescence images of the Ang II+Ad-Control group showed that the mitochondria were changed from filamentous to rounded, and the aspect ratio of the mitochondria was reduced, total area and average length of the mitochondria was reduced (Figure 4A). After transfection with the MFN2-overexpressing adenovirus, mitochondrial elongation was stimulated, total area and average length of the mitochondria was increased in the group overexpressing MFN2 compared to the Ang II+Ad-Control group. TEM showed that the mitochondria morphology of the Ang II+Ad-Control group was short and small and that the number of mitochondria was large. However, after transfection with the MFN2-overexpressing adenovirus, two short and small mitochondria underwent fusion to form large mitochondria (Figure 4B). These results indicated that overexpression of MFN2 enhanced mitochondrial fusion.

### Autophagy Inhibition Reversed the Effects of MFN2

**FIGURE 5:**
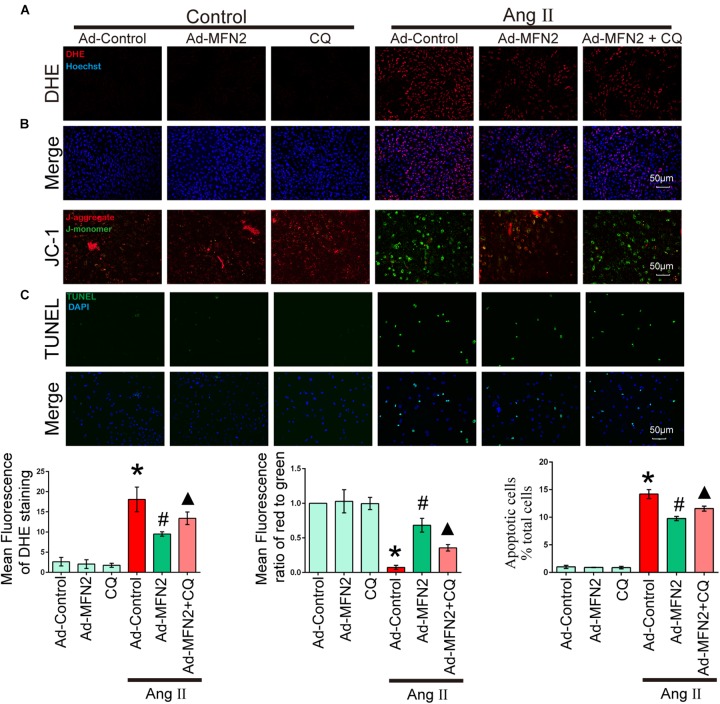
The effect of MFN2 overexpression was reversed by an autophagy inhibitor. Cardiomyocytes were divided into six groups: (1) Control+Ad-Control, cells were transfected with Control Adenovirus and were not treated with Ang II; (2) Control+Ad-MFN2, cells were transfected with MFN2 Adenovirus and were not treated with Ang II; (3) Control+Ad-MFN2+CQ, cells were transfected with MFN2 Adenovirus and treated with autophagy inhibitor CQ; (4) Ang II+Ad-Control, cells were transfected with Control Adenovirus and treated with 1 μM Ang II for 24 h; (5) Ang II+Ad-MFN2, cells were transfected with MFN2 Adenovirus and treated with 1 μM Ang II for 24 h; and (6) Ang II+Ad-MFN2+CQ, cells were transfected with MFN2 Adenovirus and treated with 1 μM Ang II for 24 h and CQ (10 μM). **(A)** DHE staining showing the oxidative stress activity of cardiomyocytes. DHE staining is shown in red, representing ROS production, and Hoechst staining in the nuclei is shown in blue. **(B)** Fluorescence images of cardiomyocytes stained with JC-1 tracker. J-monomer staining is shown in green, and J-aggregate staining is shown in red. **(C)** Cells were stained with TUNEL. TUNEL-positive cell is shown in green, and DAPI staining in the nuclei is shown in blue. ^∗^*P* < 0.05 vs. Control+Ad-Control; ^#^*P* < 0.05 vs. Ang II+Ad-Control; ^▴^*P* < 0.05 vs. Ang II+Ad-MFN2.

To determine whether MFN2 regulation of mitophagy plays a key role in Ang II-induced cardiomyocyte injury, we treated cardiomyocytes with CQ, an autophagy inhibitor. After Ang II stimulation for 24 h, cells were treated with CQ (10 μM). Cardiomyocytes were divided into six groups: (1) Control+Ad-Control, cells were transfected with control adenovirus and were not treated with Ang II; (2) Control+Ad-MFN2, cells were transfected with MFN2 adenovirus and were not treated with Ang II; (3) Control+Ad-MFN2+CQ, cells were transfected with MFN2 adenovirus and treated with the autophagy inhibitor CQ; (4) Ang II+Ad-Control, cells were transfected with control adenovirus and treated with 1 μM Ang II for 24 h; (5) Ang II+Ad-MFN2, cells were transfected with MFN2 adenovirus and treated with 1 μM Ang II for 24 h; and (6) Ang II+Ad-MFN2+CQ, cells were transfected with MFN2 adenovirus and treated with 1 μM Ang II for 24 h and CQ (10 μM). After adding CQ stimulation, the Ang II+Ad-MFN2+CQ group had higher ROS levels (Figure 5A), lower MMP (Figure 5B) and a higher apoptosis rate (Figure 5C) than the Ang II+Ad-MFN2 group. These results indicated that the effects of MFN2 overexpression, which protected against Ang II-induced cardiomyocyte injury, were reversed by CQ.

### The PINK1/MFN2/Parkin Pathway Regulates Mitophagy in Ang II-Induced Cardiomyocyte Injury

**FIGURE 6:**
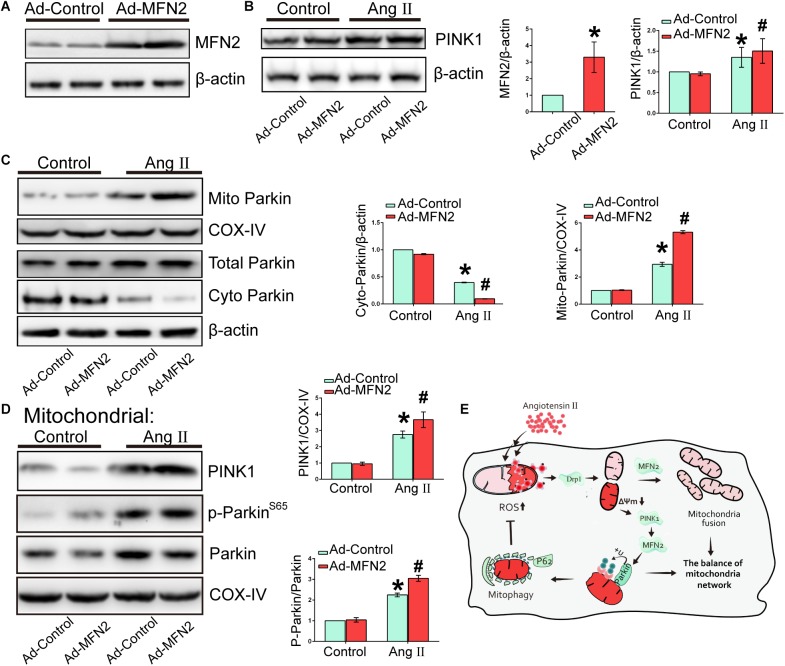
Mitofusin 2 promoted mitochondria via the PINK1/MFN2/Parkin pathway. Cardiomyocytes were divided into six groups: (1) Control+Ad-Control, cells were transfected with Control Adenovirus and were not treated with Ang II; (2) Control+Ad-MFN2, cells were transfected with MFN2 Adenovirus and were not treated with Ang II; (3) Ang II+Ad-Control, cells were transfected with Control Adenovirus and treated with 1 μM Ang II for 24 h; (4) Ang II+Ad-MFN2, cells were transfected with MFN2 Adenovirus and treated with 1 μM Ang II for 24 h; **(A)** Immunoblotting showing the overexpression of MFN2 in cardiomyocytes. **(B,C)** Immunoblotting showing the expression of PINK1, total Parkin, cyto-Parkin, and mito-Parkin. **(D)** Immunoblotting showing the expression of PINK1, Parkin, and P-Parkin in the mitochondrial fraction. Data are presented as the means ± SD (*n* = 3). ^∗^*P* < 0.05 vs. Control+Ad-Control; ^#^*P* < 0.05 vs. Ang II+Ad-Control. **(E)** Schematic model of the mechanism underlying the regulatory role of MFN2 in Ang II-induced cardiomyocyte injury.

We further tested the PINK1/MFN2/Parkin pathway and the change in the downstream protein Parkin. Cardiomyocytes were divided into four groups: (1) Control+Ad-Control, cells were transfected with control adenovirus and were not treated with Ang II; (2) Control+Ad-MFN2, cells were transfected with MFN2 adenovirus and were not treated with Ang II; (3) Ang II+Ad-Control, cells were transfected with control adenovirus and treated with 1 μM Ang II for 24 h; and (4) Ang II+Ad-MFN2, cells were transfected with MFN2 adenovirus and treated with 1 μM Ang II for 24 h. After infection with the MFN2-overexpressing adenovirus, the expression of the MFN2 protein was significantly increased (Figure 6A). Ang II stimulation significantly increased the expression of PINK1 (Figure 6B). The expression of mitochondrial Parkin (mito-Parkin) was significantly increased in the Ang II+Ad-MFN2 group compared with the Ang II+Ad-Control group (Figure 6C). Concurrently, the expression of cytoplasmic Parkin (cyto-Parkin) was decreased, and the total Parkin remained unchanged, indicating that Parkin translocates from the cytoplasm to the mitochondria. Localized to the mitochondria, Ang II stimulation significantly increased the expression of PINK1 (Figure 6D). Additionally, phosphorylation of Parkin (P-Parkin) was increased in the Ang II+Ad-MFN2 group compared with the Ang II+Ad-Control group (Figure 6D). These results indicated that MFN2 promotes Parkin translocation and phosphorylation.

## Discussion

Mitochondrial quality control plays a critical role in the development of heart failure (Ingwall, 2004; Turer, 2013; Ryan and Archer, 2015). The regulation of mitophagy-related proteins and mitochondrial fusion-related proteins can affect the progress of cardiac pathology and physiology (Hammerling and Gustafsson, 2014; Murphy et al., 2016). Moreover, protecting mitochondrial function has become a research priority in the prevention and treatment of heart failure and myocardial injury (Doenst et al., 2013; Brown et al., 2017). Using the Ang II-induced cardiomyocyte injury model, our study found that MFN2 expression was downregulated. In a study of pulmonary hypertension, Ryan et al. (2013) found that PGC-1α knockdown resulted in a decrease in Mfn2, while PGC-1α decreased after Mfn2 knockout, indicating an interaction between PGC-1α, and Mfn2. Moreover, Kackstein reported that expression of PGC-1α was decreased after 4 weeks of injection of Ang-II in mice, and skeletal muscle energy metabolism was impaired. They speculated that ROS induced the downregulation of PGC-1α (Kackstein et al., 2013). In our study, AngII induced ROS production, which was accompanied by a decrease in MFN2. A study showed a pathway between PGC-1α, ERRα, and Mfn2. PGC-1α stimulated the activity of the Mfn2 promoter, a process that required the integrity of estrogen-related receptor-α (ERRα)-binding elements (Soriano et al., 2006). The above conclusions speculated that a relationship between AngII-induced ROS, PGC-1α, and Mfn2. As we have shown, AngII stimulation led to MFN2 downregulation. We further found that MFN2 overexpression improves mitochondrial quality by regulating mitochondrial fusion and mitophagy: on the one hand, it increased mitochondrial fusion; on the other hand, it removed damaged mitochondria and maintained the energy required for the continuous mechanical work of cardiomyocytes. These processes were associated with mitochondrial fission, mitochondrial fusion and the mitophagy signaling pathway. Thus, MFN2 is critical for the regulation of Ang II-induced cardiac remodeling by regulating mitochondrial quality control.

To understand the importance of mitochondrial quality control, normal mitochondria use oxidative phosphorylation to generate energy in the form of ATP, which drives almost all biological processes, but the damaged mitochondria become a production site for ROS. An increase in ROS levels will cause a series of adverse reactions. Ang II-induced oxidative stress is critical for pathological processes in the heart (Lin et al., 2016). In particular, excessive ROS production can facilitate autophagy and apoptotic stress. In our present study, the extent of autophagy spontaneously induced by Ang II was not enough to alleviate mitochondrial dysfunction. Excessive ROS can start a chain reaction, leading to the activation of a series of signaling pathways that induce apoptosis. In this study, we also upregulated the expression of MFN2 and observed that it exerted a partial beneficial effect by reducing intracellular ROS levels and enhancing MMP in Ang II-induced cardiomyocytes relative to that in control cells. Thus, mitochondrial autophagy is responsible for alleviating intracellular oxidative stress. Similar findings have been obtained by another study, showing that autophagy protects cells against injury by mitigating intracellular oxidative stress via the IKK signaling pathway (Tang et al., 2015; Kim et al., 2016). These data demonstrate that MFN2 can reduce intracellular ROS production by regulating mitophagy. Autophagy negatively regulates oxidative stress. Moderately activated mitophagy, through the autophagic clearance of damaged mitochondria, blocks the “ROS-induced ROS release” formed by the interaction of ROS with mitochondria.

The integrity of the mitochondrial structure is extremely important for the generation of cellular energy and the maintenance of cardiac function (Murphy et al., 2016; Brown et al., 2017; Melenovsky et al., 2017). A large number of studies have shown that normal mitochondrial function depends on normal mitochondrial dynamics (Song et al., 2015). Mitochondria are dynamic organelles that undergo fission and fusion, constantly moving along microtubules to where energy is most needed. These processes are necessary for normal mitochondria and the mitochondria-intensive distribution of cardiomyocytes. Numerous studies have shown that the loss of fusion and fission proteins is fatal to cells (Youle and van der Bliek, 2012), but the exact underlying mechanism remains unclear. Therefore, we focused on mitochondrial dynamics. Mitochondrial fusion is the transformation of two mitochondria into one larger mitochondrion. There are three proteins involved in the process of mitochondrial fusion. The mitochondrial outer membrane fusion protein is MFN1/2, and the synthesis of the inner membrane depends mainly on OPA1. OPA1 maintains a tight junction of the intima and prevents the leakage of cytochrome C. Chen et al. (2010) demonstrated the necessity of mitochondrial fusion proteins for maintaining mitochondrial DNA stability in skeletal muscle cells. Moreover, an *in vivo* experimental study found that depletion of MFN2 would lead to cell apoptosis (Peng et al., 2015). Our experiments showed that after Ang II stimulation, the expression of MFN2 decreased, the mitochondria were fragmented, ROS levels were increased, the apoptosis rate were increased and the MMP were decreased. After overexpressing MFN2, the morphology of the mitochondria changed. Fluorescence images of mitochondria show that they change from punctate to elongated; Furthermore, ROS and apoptosis rate were decreased, and MMP was increased. Based on these findings, we demonstrate that MFN2 participated in mitochondrial fusion against AngII-induced cardiomyocyte injury.

Mitofusin 2, a mitochondrial dynamics-related protein, has other functions that are more important than mitochondrial fusion, such as autophagy, which removes damaged mitochondria. This process prevents mitochondria from undergoing unnecessary fission and fusion. MFN2 is a mitochondrial outer membrane fusion protein and Parkin ubiquitination substrate (Gegg et al., 2010). MFN2 is abundantly expressed in the cardiomyocyte cytoplasm and mitochondria and regulates mitochondrial homeostasis by regulating mitochondrial autophagy signaling. The PINK1-MFN2-Parkin signaling pathway plays a critical role in regulating mitophagy. Chen’s group reported that in genetically normal cardiomyocytes, mitochondrial uncoupling with FCCP resulted in Parkin redistribution into intracellularly mitochondria-rich regions, whereas in MFN2-depleted cells, Parkin diffused in the cytoplasm, indicating that endogenous MFN2 promotes the need for Parkin localization to depolarize mitochondria. Strategies to increase mitophagy can improve cardiomyocyte mitochondrial quality. Although the increased mitophagy activity at stage of cardiomyocyte progressive injury is physiologically adaptive, the magnitude and impact of this adaption can be significantly limited by some factors. Our study show that in the Ang II stimulation model, elevated autophagy activity was accompanied by high levels of oxidative stress and apoptosis, and this physiological adaptation could not improve the injury caused by AngII. Due to the consumption of MFN2 was not sufficient to promote more Parkin to translocate to the mitochondria. Moreover, with the consumption of MFN2, the mitochondrial quality control pathway of PINK1-Parkin is disrupted, leading to an accumulation of mitochondrial abnormalities and cardiotoxicity. We found that overexpression of MFN2 promoted Parkin translocation and phosphorylation and upregulated the expression of autophagy-related proteins in the Ang II model, thereby regulating mitophagy to clear damaged mitochondria. However, the effects of MFN2 overexpression, which protected against Ang II-induced cardiomyocyte injury, were reversed by CQ. MFN2-induced mitophagy interrupted the chain reaction caused by the damaged mitochondria, prevented the amplification of oxidative stress and prevented cardiomyocyte injury. Moreover, MFN2 promoted mitochondrial fusion and improved mitochondrial quality. Thus, MFN2 Participated in mitophagy and mitochondrial fusion against AngII-induced cardiomyocyte injury.

## Conclusion

As shown in the mechanism diagram (Figure 6E), this study demonstrates that MFN2 promotes Parkin translocation and phosphorylation, leading to mitophagy to clear damaged mitochondria. Concurrently, MFN2 participates in mitochondrial fusion to maintain mitochondrial quality. Taken together, these results indicate that MFN2 mediates both mitophagy and mitochondrial fusion in Ang II-induced cell injury and that regulation of MFN2 and PINK1/MFN2/Parkin pathway-mediated mitophagy and mitochondrial fusion may has a compensatory, protective role in Ang II-induced injury.

## Ethics Statement

All procedures were approved by the Animal Care and Use Committee of Southern Medical University. The investigation conformed to the Guide for the Care and Use of Laboratory Animals published by the US National Institutes of Health (NIH Publication No. 85–23, revised 1996).

## Author Contributions

WX, HR, and DX conceived, designed, and coordinated the study. WX, ZM, DA, ZL, and WC participated in the experiments and carried out the data analysis. WX drafted the manuscript. YB and QZh involved in the data interpretation and revised the manuscript. WL and QZe involved in the study design.

## Conflict of Interest Statement

The authors declare that the research was conducted in the absence of any commercial or financial relationships that could be construed as a potential conflict of interest.

## Supplementary Material

FIGURE S1 overexpression of MFN2 affected Drp1.

## Abbreviations

Ang II: angiotensin II
LC3B: microtubule-associated protein 1 light chain 3 beta
MFN2: mitofusin 2
MMP: mitochondrial membrane potential
PINK1: PTEN induced putative kinase 1
ROS: reactive oxygen species
