# Supplementary figures and images for: Mitofusin 2 Participates in Mitophagy and Mitochondrial Fusion Against Angiotensin II-Induced Cardiomyocyte Injury
Source: Front Physiol. 2019 Apr 10;10:411. doi: 10.3389/fphys.2019.00411 (PMC6468045; doi:10.3389/fphys.2019.00411)

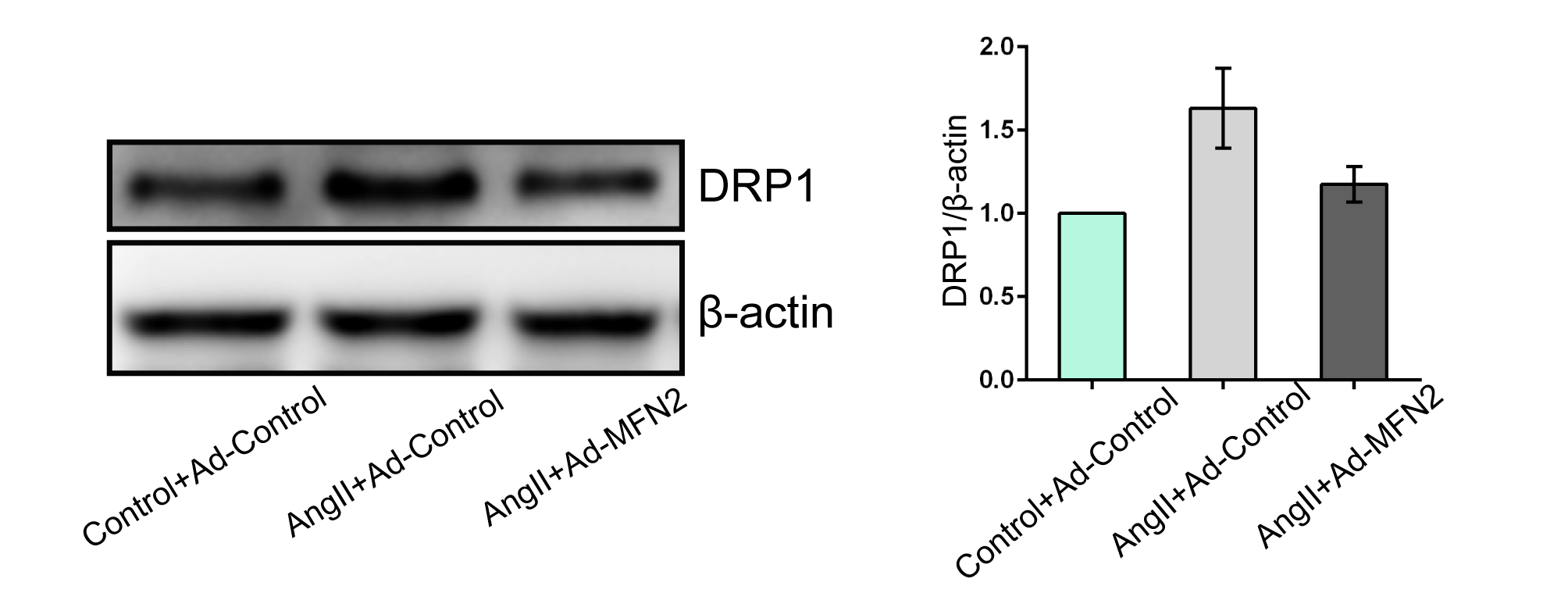

Supplement: FIGURE S1 — overexpression of MFN2 affected Drp1. [file Image_1.TIF]
